# Controversies in autism: is a broader model of social disorders needed?

**DOI:** 10.1186/1753-2000-7-9

**Published:** 2013-03-18

**Authors:** Michal Hrdlicka, Iva Dudova

**Affiliations:** 1 Department of Child Psychiatry, Charles University Second Faculty of Medicine and University Hospital Motol, Prague, Czech Republic

**Keywords:** Autism, Recovery, Oxytocin, Early deprivation, Herpes encephalitis, Preterm children, Social phobias, Personality disorders, Autistic traits, Social inhibition disorders

## Abstract

This article examines the most significant, contradictory evidence pertaining to autism. The first section of the article includes reports of recovery from autism, data obtained from studies involving oxytocin, early deprivation, autism in preterm children, late-onset autism, and symptom overlap among ASD, social phobias and personality disorders. In the second section of the article, we offer a model that better incorporates current findings and address controversies that continue to surround ASD. We propose an umbrella term “social inhibition disorders” which integrates autism spectrum disorders and social phobias, as well as schizoid, schizotypal, and obsessive-compulsive personality disorders. It would also include “quasi-autism,” which has been found in early deprivation studies, autism in preterm children, and cases of late-onset autism presenting after herpes encephalitis infection. Finally, we discuss suggestions for further research and clinical perspectives.

## Introduction

According to the Diagnostic and Statistical Manual of Mental Disorders, Fourth edition, Text revision (DSM-IV-TR) and the International Classification of Diseases, Tenth edition (ICD-10), autism spectrum disorders (ASD) are characterized by severe and pervasive abnormalities in reciprocal social interaction and communication skills, and the presence of stereotyped behaviors, interests and activities which lead to life-long impairments [1,2]. There is broad consensus that ASD belongs to the category of neurodevelopmental disorders and this opinion is based strongly on neurobiological factors [3-6]. There is no doubt about the genetic basis of autism and, from the etiological point of view, it is distinguished from syndromic autism (connected with known causes, such as tuberous sclerosis, fragile X-syndrome and rare genetic syndromes) and non-syndromic autism that constitutes the majority of observed ASD cases [7,8].

The proposed DSM-5 manual combines Autistic Disorder, Asperger Disorder, Childhood Disintegrative Disorder and Pervasive Developmental Disorder – Not Otherwise Specified (PDD-NOS) into a newly re-categorized description of Autism Spectrum Disorder [9,10]. Three symptom domains (social, communication, and repetitive behavior) are condensed into two domains (social-communication and repetitive behaviors). The new proposal also eliminates delayed language development as a symptom (i.e. it is not specific to ASD) [10]. Additionally, the new changes emphasize disturbed social development as the major clinical hallmark of autism [11].

The sheer volume of recent publications on autism clearly indicates its position as a prominent topic in pediatric psychiatry. For example, Hughes found 1300 reports on autism published in 2008 [12] and the number has continued to steadily increase. As the number of publications continues to increase, the number of cases that contradict the codified concept of autism continues to rise. The first objective of this article is to examine the most significant, contradictory evidence pertaining to autism. Included are reports of recovery from autism, data obtained from studies involving oxytocin, early deprivation, autism in preterm children, late-onset autism, and symptom overlap among ASD, social phobias and personality disorders. The second objective of the article is to propose a model that better incorporates current findings into a unified concept and better addresses the controversies surrounding the subject.

## Recovery from autism

Autistic Spectrum Disorders (ASD) have generally been regarded as life-long conditions. However, in recent years it has been claimed that a significant minority of children, with well-documented ASD, have recovered from the disorder [13,14]. Helt et al. [13] described the phenomenon in terms of “recovery”, “best outcome” and “optimal outcome”. Helt et al. also suggested a definition that fulfills these terms [13]. From a historical perspective, the definition includes a child having been diagnosed, by a specialist, in early childhood (i.e., by the age of 5 years), the presence of language delay and an analysis of early reports and/or home videotapes which support the diagnosis. From a present-day perspective, the definition states that the subject does not meet the criteria for any ASD, does not meet any ASD cut-off on the Autism Diagnostic Observation Schedule (ADOS) [15], and assessment results in various aspects of everyday functioning are positive.

The first study mentioning the possibility of individuals with ASD “losing” the ASD diagnosis was presented by Rutter [16]. His early longitudinal outcome study reported that 1.5% of the original group was functioning normally at the time of follow-up. Lovaas [17] reported that 9 of 19 children (47%) who had undergone intensive behavioral therapy had achieved normal intellectual and educational functioning compared to only 2% of the control group. In a long-term follow-up, Sigman & Ruskin [18] found that 10% of 51 children had “lost” their ASD diagnoses over time. Seltzer et al. [19] described that 11.9% of their sample (n = 405), which was originally diagnosed as having Autistic Disorder, no longer met any of the criteria for an ASD diagnosis when (current) Autism Diagnostic Interview-Revised (ADI-R) [20] scores were applied.

Zappella [21] reported that 7.3% of a sample of 534 cases “outgrew” their autism and fully recovered their intellectual and social abilities. Nearly all of the autistic children who recovered (36 of 39) had a history of autistic regression. Interestingly, 70% of the “recovered children” also suffered from ADHD, and 56% suffered from persistent tics.

Pellicano [22] examined 37 children with ASD diagnosed using the ADI-R (mean age = 5.6 years). Three years later, the children were re-assessed using the ADOS. It was found that 19% of children could no longer be diagnosed as having ASD; of special note, this group comprised children who began receiving behavioral intervention from a very young age – significantly earlier than those children who experienced less improvement over time (28 vs. 42 months). The study is also unique in that it was the only study where the original diagnosis was determined using the diagnostic “gold standard instrument,” i.e. the ADI-R. Re-assessment using the ADOS (as recommended by Helt et al. [13]) also precisely noted the age at first diagnosis and age of initiation of behavioral therapy. Absence of such an exact methodology in previous studies allowed theoretical doubts regarding whether all of the children who “lost” their ASD diagnosis had originally been correctly diagnosed.

In their comprehensive review, Helt et al. [13] found that 3 – 25% of children reportedly “lost” their ASD diagnoses and attained a normal range of cognitive, adaptive and social skills. Predictors of recovery included relatively high levels of intelligence, receptive language skills, verbal and motor imitation, and motor development. Early diagnosis and treatment were associated with a better prognosis, as was a diagnosis of PDD-NOS. The presence of seizures, mental retardation and genetic syndromes were clearly unfavorable signs.

The most recent study on developmental trajectories utilized a different methodological approach but the results were similar. Fountain et al. [23] identified 6 developmental trajectories in their sample of 6975 autistic children aged 2 to 14 years. One group representing approximately 10% of the children experienced rapid gains, advancing from severely affected to high functioning. Studies such as the ones cited above, which describe recovery from autism, clearly question the paradigm of ASDs as life-long conditions.

## Oxytocin studies

Oxytocin (OT) is a hormone synthesized in the hypothalamus. It facilitates parturition and lactation. It is also associated with the development of prosocial behavior, such as mother-infant attachment, grooming, approach behavior, sexual activity, and stress regulation. Studies in healthy volunteers have suggested that OT increases trust and cooperation as well as boosts social perceptiveness, such as face recognition and the ability to read what is on someone´s mind from the look in their eyes [24].

Eric Hollander was the first to perform oxytocin trials involving ASDs. In the initial study, the frequency of repetitive behaviors decreased during OT infusions compared to placebo infusions [25]. In a later study, he reported that individuals with ASD who received OT infusions experienced long-term (2 week) improvement in comprehension of affective speech, whereas placebo effects were only short-term [26].

Recent studies have also been positive. OT inhalation was found to enhance interactions with others, as well as feelings of trust and preference. Additionally, it selectively increased the duration of gaze directed towards the eyes, which is thought to be a prosocial effect [27]. In other studies, intranasal OT administration was described as having improved the ability of ASD subjects’ to recognize the emotional states of others [28] and having improved measures of social cognition and quality of life [29].

If the codified concept describing ASDs as disorders with severe, pervasive abnormalities in reciprocal social interaction and life-long impairment is unequivocally true, then the interpretation and explanation of oxytocin studies become problematic at best. Andari et al. [27] suggested that patients with autism might possess latent social skills, and thus oxytocin might favor social engagement behavior by suppressing fear and mistrust. This explanation is both reasonable and rational; however, the concept of latent social skills certainly represents a spanner in the works of the current prevailing opinion on autism.

## Early deprivation studies

For quite some time, the only evidence available on this subject was derived from rare case studies. The opportunity to systematically examine the psychological effects of early global deprivation first arose when Western countries (such as the United Kingdom and the Netherlands) facilitated the adoption of large numbers of Romanian children who had been raised under impoverished conditions in deplorable institutions following the collapse of the Ceaucescu regime [30].

Rutter and his team longitudinally followed 144 adopted Romanian children who arrived in the UK before the age of 42 months. Detailed assessments were performed at 4, 6, and 11 years of age and the results were compared with a sample of 52 domestic adoptees who had not be early-reared in an institution [31]. Sixteen children were found to have quasi-autistic patterns confirmed by the ADI-R and ADOS; a rate of 9.2% in the Romanian institution-reared adoptees (IQ of at least 50), compared to a rate of 0% in the domestic adoptees. A follow-up of the children showed that a quarter of the children had “lost” their autistic-like features by age 11.

Similar results were demonstrated in the Netherlands. Hoksbergen et al. [32] used the [33] Auti-R scale to study 80 Romanian adoptees with a median age of 8 years. Thirteen of the 80 children (16% of the group) scored within the autistic range. The sex ratio was approximately equal, a result similar to the British adoptee studies, but in sharp contrast to the male preponderance which is characteristic of autistic samples. Hoksbergen et al. used the phrase “post-institutional autistic syndrome,” a term which could be considered essentially equivalent to Rutter’s “quasi-autism.”

These early deprivation studies challenge the paradigm of ASDs as neurodevelopmental disorders with a neurobiological basis, and indicate a need to develop a more complex vulnerability theory that can also integrate extreme psychological factors into the possible causative mechanisms. Moreover, epigenetic factors could play a role here. It has been hypothesized that nutrient – gene interaction, encompassing various genetic and environmental factors such as dietary folate and vitamin B intake, amino acid deficiencies, and environmental exposures, could modify expression of certain metabolic pathways [34]. All of these environmental options could have been involved in the cases of malnourished Romanian adoptees initially reared under the abysmal hygienic conditions associated with orphanages during the Ceaucescu regime.

## Preterm children

There is emerging evidence suggesting that low birth weight and prematurity may also be a risk factor for ASDs. Recent studies on these topics described the prevalence of ASDs among prematurely born children being in the range of 3.65 – 8%. Hack et al. [35] screened 219 preterm-children (birth weight < 1000 g) at 8 years of age, and compared them with 176 term-children of similar maternal sociodemographic status, sex, and age. The Parent Child Symptom Inventory (CSI-4) was utilized and 8 subjects (3.65%) with ASD were identified in the preterm group, compared to 1 child (0.57%) in the control group. In a British study, 219 preterm-children (< 26 weeks of gestation) were screened at 11 years of age and compared with 153 term-children [36]. The Social Communication Questionnaire (SCQ) was utilized and an ASD diagnosis was confirmed in 8% of the preterm-children, compared to 0% of the controls. The only study in which highly reliable diagnostic instruments (such as the ADI-R or ADOS) were used, was a study performed by Pinto-Martin et al. [37]. Preterm-children (birth weight < 2000 g) were screened at 16 years of age and then clinically examined at age 21. The percentage of those with ASD was calculated to be as high as 5% of the regional preterm birth cohort.

Prematurity seems to be rather a non-specific risk factor compared to the specific risk factors for autism that have been previously suggested, such as tuberous sclerosis, fragile X-syndrome and certain rare genetic syndromes [7,8]. Losh et al. [38], in a same-sex twin study, estimated that every 100 g increase in birth weight showed a 13% reduction in the risk of ASD. Biological vulnerability factors in preterm children seem to be obvious. Additionally, there is developing evidence that psychological factors are also important. Smith et al. [39] presented evidence from a sample of 44 children born at < 26 weeks of gestation. Magnetic resonance imaging (MRI) revealed that the number of stressors, to which an infant was exposed, to be directly associated with decreased frontal and parietal brain width, altered diffusion measures and functional connectivity in the temporal lobes; additionally, increased abnormalities in motor behavior were observed during neurobehavioral examinations. The study by Smith et al. is yet another indication that a more complex and comprehensive vulnerability theory for ASDs is needed.

## Late onset of autism

Early onset is a significant factor in ASD. With regard to diagnosing either Autistic Disorder (DSM-IV-TR) or Childhood Autism (ICD-10), delayed or abnormal function must be present before the age of 3 years. In fact, early-onset has become a general assumption for the vast majority of ASDs. This assumption is in agreement with the model of ASDs as neurodevelopmental disorders with early brain overgrowth and dysfunction. Existing research suggests that autistic individuals have larger brains volumes, cerebellums, and caudate nuclei; however, the area of the corpus callosum is reduced. Results from studies involving the amygdala and hippocampus volumes in autistic subjects remain inconsistent and no changes have been detected in thalamic volume [40,41].

The only routine exception from the early onset paradigm is childhood disintegrative disorder (Heller’s syndrome), a rare disorder linked to the ASD family. The DSM-IV-TR criteria state that the disorder begins between 2 – 10 years of age, whereas the ICD-10 criteria have the same lower limit but do not include any upper age limit. Malhotra & Gupta [42] found that the average age of onset was 3.76 years (range 2 – 8.75), and precipitating factors were significantly more frequent in childhood disintegrative disorder than in typical autism (50% vs. 19%).

In addition to the above cases, other sources regarding unconventional ages of ASD onset have emerge in the form of rare case reports describing the onset of autism in late childhood [43,44] and adolescence [45]. There have even been reports of adulthood onset autism [46]; however, regardless of age, these late onset presentations are typically associated with herpes encephalitis infections. While marked signs of general cognitive decline appeared shortly after the encephalitis, autistic symptoms appeared weeks to months following the infection. Some of the referred patients fulfilled the diagnostic criteria of the DSM edition (valid at the time of examination; DSM-III-R, DSM-IV) with the notable exception of the onset criterion; however, formal/specific information was lacking in other cases. This variation prompted Gillberg [46] to suggest that autism was not necessarily a developmental disorder in the sense that it was only capable of presenting in typical form during early development.

The philosopher Karl Popper [47] put it very succinctly, no number of positive outcomes at the level of experimental testing or observation can confirm a scientific theory, but a single counterexample is logically decisive: it shows the theory, from which the implication was derived, to be false. Although the Popper´s maxim sounds too strict for neurobehavioral science, the above-mentioned case reports regarding late-onset autism may very well destabilize the paradigm of early-onset ASDs.

## Overlap of ASDs with social phobia and some personality disorders

The DSM-IV-TR states that fear and avoidance in social phobia (SP) should not be better accounted for by other mental disorders (e.g., pervasive developmental disorder, PDD), but research suggests that ASDs and SP can occur concurrently [48]. There is mounting evidence supporting the existence of comorbidity between high-functioning autism (HFA) and SP. Individuals with HFA report SP symptoms at a rate ranging from 11.7 – 57.1% [48]. The co-occurrence of both conditions has been known to appear not only in young adults, but in children and adolescents as well [49]. However, further research is needed to determine if HFA and SP do, in fact, follow different trajectories for social skills development as current, but separately conducted, studies suggest [48].

Lugnegard et al. [50] stressed that the relationship between DSM-IV Personality Disorders (PD) and PDD/ASD was not completely clear. Although presently classified as an Axis I disorder, the basic characteristics of PDD/ASD (e.g. pervasive impairment, abnormal development) are, in fact, equal to those of Axis II disorders (e.g. the pattern is stable and of long duration, and its onset can be traced back at least to adolescence or early adulthood). Furthermore, researchers wonder whether temperament and personality, as viable concepts for the study of typical development, can be applied to the study of clinical syndromes such as autism [51].

Research on similarities and overlap between ASD and PD has been limited. Hurst et al. [52] administered specific questionnaires to a large non-clinical adult sample of 607 college students. They found the Asperger´s and Schizotypal questionnaires were positively correlated. Barneveld et al. [53] compared a group of 27 adolescents with ASD to 30 typically-developing adolescents. Within the ASD group, 11 adolescents (40.7%) satisfied the DSM-IV-TR criteria for schizotypal personality disorders, compared to 0% in the control group. Lugnegard et al. [50] examined 54 young adults diagnosed with Asperger syndrome. Approximately half of the study group met the criteria for a personality disorder: 14 participants (26%) for schizoid PD, 10 (19%) for obsessive-compulsive PD, 7 (13%) for avoidant PD, and 1 (2%) for schizotypal PD. Based on these results, the authors questioned the existence of a “pure” schizoid PD without a concomitant PDD.

## A broader model for ASDs: social inhibition disorders?

Dawson and others have proposed a social motivation hypothesis of autism. This hypothesis suggests that some impairment evident in ASDs, such as the well-documented face-processing impairment, are not fundamental, but are secondary to the fundamental impairment in social motivation. As a result of reduced social motivation, the infant at risk for ASD spends less time paying attention to, and socially engaging with, other people. Reduced social engagement with the “world” contributes to a failure to develop expertise in processing face, language and other elements of social information exchange. Because experience drives cortical specialization, reduced attention to “others” results in a failure of specialization and less efficient function of the brain regions that mediate social cognition [54]. Indeed, hypo-activation of the fusiform gyrus and amygdala in autistic individuals during face perception tasks has been repeatedly found in well-designed functional MRI studies [55].

The social motivation hypothesis can also assimilate the results of early deprivation studies into the concept of autism, as well as those of preterm children. All of the factors which negatively interfere with the development of social expertise can be viewed, in a broader sense, as vulnerability factors. One potential lesson stemming from the oxytocin studies is that at least some autistic children may possess latent social skills [27] which can be unmasked and revealed through oxytocin administration. This leads to a cardinal question: wouldn’t this hypothesized subgroup of children be nearly identical to the group of children who experienced “optimal outcomes” in the recovery studies?

**Figure 1:**
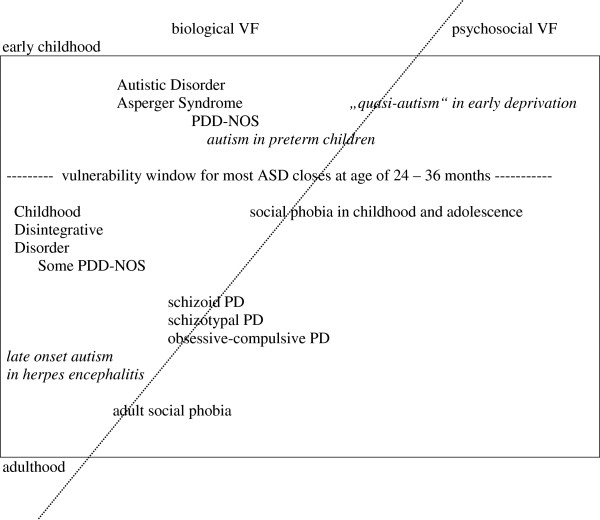
**Social inhibition disorders.** This figure presents the conditions included under this suggested umbrella term as well as the relationships between the included conditions, age and vulnerability factors. VF – vulnerability factors, PDD-NOS - Pervasive Developmental Disorder – Not Otherwise Specified, ASD - autism spectrum disorders, PD - personality disorder.

Furthermore, it seems plausible to take one further step ahead and consider a broader model of social disorders in psychiatry. Our suggestions are demonstrated in Figure 1. We propose the umbrella term “Social Inhibition Disorders.” Within this proposed concept, we hypothesize that all ASDs, as well as some other social disorders, could generally be viewed as the brain functioning in a “socially reduced mode” or “socially safe mode” (if we may express it symbolically using Microsoft Windows terminology) in response to a variety of more or less specific damaging, overloading and long-lasting conditions. The presentation of the “socially safe mode” state of Social Inhibition Disorders would depend upon age and developmental stage and would manifest in variety of social symptoms that are partially common (e.g. social inhibition and social clumsiness) and partially unique for each diagnosis grouped under the umbrella of “Social Inhibition Disorders.”

The “vulnerability window” for most ASDs, which can be derived from studies on autistic regression, seems to close during the toddler years, given that the average onset of regression is consistently described as being between 14 and 24 months of age [56]. That having been said, rare cases of herpes encephalitis have demonstrated that the “biological vulnerability window” for ASD can be “re-opened” as a repercussion of serious organic brain damage in late childhood, adolescence or even adulthood.

Milder reductions in social motivation and expertise (in terms of Dawson´s social motivation hypothesis), which may be conditioned on a lower grade of biological vulnerability factors, does not lead to true ASD, but could manifest as social phobia or schizoid, schizotypal, or obsessive-compulsive personality disorders at a later age. The connection between these disorders and non-syndromic ASDs could be the concept of continuously distributed autistic traits, which was introduced to autism research by John Constantino and coworkers.

Constantino & Todd [57] examined 788 sets of twins aged 7 to 15 years and found that autistic traits, as measured using the Social Responsiveness Scale, were common, continuously distributed and moderately to highly heritable. Levels of severity of autistic traits at or above the previously published means for patients with PDD-NOS were found in 1.4% of boys and 0.3% in girls. The hypothesis that the variation in autistic traits could be attributed to a single continuously distributed underlying factor was later confirmed in a clinical sample of patients with ASD and other psychiatric disorders [58] and in siblings of children with ASD [59]. Robinson et al. [60] studied 5968 sets of twins aged 12 years using the Childhood Autism Spectrum Test (CAST). Moderate to high heritability was found for autistic traits in the general population (53% for females and 72% for males). There were no differences in heritability between extreme groups and the general population. The data provided support for a continuous risk hypothesis and for conceptualizing ASD as the quantitative extreme of a neurodevelopmental continuum.

To our knowledge, there have yet to be any specific studies focused on autistic traits in patients with schizoid, schizotypal and obsessive-compulsive personality disorders or social phobia. In our opinion, it is an essential issue that deserves intensive research in the near future.

Indirect observations were made by Hallett et al. [61] when the authors studied approximately 6000 sets of twins ages 7 and 8 and again at age 12 using CAST (for identifying autistic-like traits) and the emotional problems subscale of the Strengths and Difficulties Questionnaire (for measurement of internalizing traits). It was found that autistic-like traits at age 7 made a modest but significant contribution to the presence of internalizing traits at age 12. There was also an association between shared environmental influences on the two traits, particularly at ages 7 and 8. It suggests that environmental factors, such as parental and home influences, may be important. The observation supports the role of psychosocial vulnerability factors, which are suggested in our model (see Figure 1).

Heterogeneity within the autism spectrum is, perhaps, the single greatest obstacle to research at all levels [62]. The overlap of ASDs with other social disorders seems to contribute to the confusion in autism research. Our model offers an unambiguous solution to overcome this handicap.

## Conclusions

The inclusiveness and comprehensiveness of the Social Inhibition Disorders concept could lead to the following research and clinical advantages:

1. It enables a closer study of the connections among autism, social phobia and some PDs. It lets us ask, “Is it simply comorbidity, or is there a developmental transition in some cases?”

2. It help integrate the study of shared features and vulnerability factors among these social disorders, as well as allowing closer study of the interaction between biological and psychosocial vulnerability factors.

3. It facilitates the complex study of autism recovery cases.

4. It could also facilitate basic autism research by minimizing the need to hammer the proverbial square peg into a round hole, which was so aptly expressed in the title of the article “Time to give up on a single explanation for autism”, Happe et al. [63].

## Abbreviations

ADI-R: Autism Diagnostic Interview-Revised; ADOS: Autism Diagnostic Observation Schedule; ASD: Autism spectrum disorders; DSM-IV-TR: Diagnostic and Statistical Manual of Mental Disorders, Fourth edition, Text revision; HFA: High-functioning autism; ICD-10: International classification of diseases, 10th revision; MRI: Magnetic resonance imaging; OT: Oxytocin; PD: Personality disorders; PDD: Pervasive developmental disorders; PDD-NOS: Pervasive developmental disorder – not otherwise specified; SP: Social phobia

## Competing interests

The authors declare that they have no competing interests.

## Authors’ contributions

MH was responsible for writing the manuscript. ID commented on the written drafts of the manuscript. Both authors read and approved the final manuscript.
